# A Single-Center Experience in Combined Oncological–Surgical Treatment for Resectable Locally Advanced Non-Small Cell Lung Cancer (NSCLC)

**DOI:** 10.3390/diseases12050098

**Published:** 2024-05-12

**Authors:** Dan Levy Faber, Abed Agbarya, Ben Caspy, Moshe Lapidot, Shoshana Keren Rosenberg, Sonia Schneer, Erez Sharoni, Ronen Galili

**Affiliations:** 1 Department of Cardiothoracic Surgery, Lady Davis Carmel Medical Center, 7 Michal St., Haifa 3436212, Israel; ben@clalit.org.il (B.C.); sonias3@clalit.org.il (S.S.); esharoni@clalit.org.il (E.S.); ronenga@clalit.org.il (R.G.); 2 Rappaport Faculty of Medicine, Technion-Israel Institute of Technology, Haifa 3109601, Israel; shoshanakr@clalit.org.il; 3 Oncology Institute, Bnai-Zion Medical Center, Haifa 3339419, Israel; 4 Azrieli Faculty of Medicine, Bar Ilan University, Tzfat 3436212, Israel; moshel@gmc.gov.il; 5 Department of Thoracic Surgery, Galilee Medical Center, Nahariya 2210001, Israel; 6 Oncology Institute, Lin Medical Center and Carmel Medical Center, Haifa 3515210, Israel; 7 Pulmonary Division, Lady Davis Carmel Medical Center, Haifa 3436212, Israel

**Keywords:** locally advanced stage, neoadjuvant treatment, non-small cell lung cancer, surgery, chemotherapy, radiotherapy, immunotherapy

## Abstract

Non-small cell lung cancer (NSCLC) is the most common pulmonary malignancy, frequently diagnosed at an advanced stage (III/IV). Patients in the Locally Advanced Stage Subgroup (IIIA) are relatively few, yet compose heterogenic phenotypes, posing a diagnostic and treating challenge, leading to a lack of clinical guidelines regarding the optimal standard of care. Several approaches exist, with a general agreement that a combined oncological and surgical modality approach is required. In this current retrospective descriptive study, patients with operable stage IIIA NSCLC who underwent surgery between 2013 and 2020 were evaluated on several aspects, including the initial diagnosis, neoadjuvant regimens, outcomes of surgical intervention, and overall survival at 2 years and 5 years following treatment. A total of 35 patients had neoadjuvant oncological treatment (mostly chemoradiation therapy) prior to surgery, out of which 28 patients were diagnosed with stage IIIA NSCLC. In post-operative assessment of pathological staging, downstaging was reported in 19 patients, of which 25% of cases were defined as a complete pathological response. The 2-year overall survival rate was 65% and the 5-year overall survival rate was 62%. The main pattern of disease recurrence was distant metastasis.

## 1. Introduction

Lung cancer today is the second most common malignancy and the leading cause of cancer death for both sexes worldwide [1,2,3,4]. According to the Surveillance, Epidemiology, and End Results (SEER) database, the estimated number of new lung cancer cases for 2023 in the United States was 238,340 with 127,070 deaths [5]. There are two histological types of lung cancer, namely small cell lung cancer, which accounts for 15% of the cases, and non-small cell lung cancer (NSCLC), which dominates in the majority of the diagnosed cases [3,6,7]. NSCLC subtypes are adenocarcinoma, large-cell carcinoma, and squamous cell carcinoma [6].

The determination of different stages pertinent to this disease is performed by considering the primary tumor (T) location, its size, infiltration to lymph nodes (N), and the presence of metastases (M) according to TNM classification, which details each category [6,8].

Early stages of lung cancer (I, II) are detected in 32% of cases. The 5-year survival rate for this group of patients reaches as high as 92% in stage IA1 and 53% in stage IIB [8,9].

Nevertheless, a further advanced stage (III), which is considered to be locally involved, has a 5-year survival rate as low as 36 to 13%.

More than 70% of all new lung cancer cases are diagnosed in an advanced stage, either locally advanced/regional (stage III) or metastatic (stage IV) [5]. These clinical presentations have an unpromising prognosis [6] because of late diagnosis timing. Life expectancy of patients with lung cancer is relatively poor due to the association of the disease detection with the characterized progressed stage [6].

The stage of locally advanced disease is very heterogenic [4] and according to the eighth edition of the lung cancer staging system, it is composed of three subgroups (IIIA, IIIB, and IIIC). Subgroup IIIA includes patients with a tumor up to 5 cm with mediastinal lymph node involvement, patients with a larger tumor size (>5 cm) with hilum lymph node involvement, and patients with a tumor size larger than 7 cm without lymph node involvement. Subgroup IIIB includes patients with a tumor up to 5 cm and contralateral lymph node involvement or patients with a larger tumor size (>5 cm) and mediastinal lymph node involvement. Subgroup IIIC includes patients with a tumor size larger than 5 cm and contralateral lymph node involvement [10].

Patients in the metastatic stage are referred to definitive oncological treatment, which includes chemotherapy, radiotherapy, and today also biological and immunological therapy for suitable patients [11,12].

Treatment recommendations for this complex stage are debatable. According to the National Comprehensive Cancer Network (NCCN) guidelines, patients in the stage IIIA group (T1-2 N2, T3 N1, T4 N0-1) with resectable disease should be evaluated for surgical resection after the induction of systemic oncological therapy with or without radiotherapy and with no apparent tumor progression during neoadjuvant therapy [11]. For a higher stage of locally advanced disease (IIIB, IIIC with the exception of selected T3 noninvasive N2-positive cases), the recommendation is for definitive oncological treatment.

Several databases estimate that 20–35% of patients with lung cancer are diagnosed at stage III [13]. A recent study from Spain, based on the thoracic tumor registry, demonstrated that 28.4% of patients with lung cancer presented with stage III of which 15.8% were at stage IIIA, 11.6% at stage IIIB, and 1% at stage IIIC [14]. Survival data of this lung cancer stage are problematic due to the heterogenicity of this group of patients. The SEER database analyzed 4564 cases of unresectable stage III lung cancer (2009–2014) and found that the overall survival was 14.8 months with radio-chemotherapy and 13.2 months with chemotherapy. An overall 5-year survival rate of 34.5% was calculated for patients with regional lung cancer (2009–2015) [5,15,16]. Patients with resectable locally advanced lung cancer disease seem to have better overall survival rates, reaching as high as 27–34 months [16].

Treatment modalities of non-small cell lung cancer have evolved over the years [3,17,18]. Historically, surgery was offered as a standard treatment for patients with operable lung cancer to remove the tumor [4]. Initially, in 1930, it was performed as a pneumonectomy procedure, and a couple of decades later, in 1950, lobectomy became oncologic gold standard resection [4]. However, upon follow-up at a median of 4.5 years, recurrence was observed for 70% of patients diagnosed with stage IB-IIIA. Loco-regional and distant metastases were found for 33% and 67% of cases [19].

In the search for better outcomes, e.g., to prolong survival, and to prevent relapse of lung cancer, scientific investigations suggested the administration of chemotherapy post-surgery [3,20]. This approach showed a mere increase in the overall survival rate by 5% upon the administration of platinum-based chemotherapy of cisplatin or carboplatin [21].

Adjuvant therapy following surgery has tried to augment its effect through the addition of radiotherapy [4], e.g., stereotactic body radiation therapy (SRBT), as part of the oncological treatment of patients with NSCLC. A radiotherapy total dose of 55 Gy was delivered in 20 daily fractions [22], which resulted in a non-proven benefit for patients upon comparison to a treatment protocol without radiotherapy [23]. The postoperative radiotherapy (PORT) was recommended by NCCN only for patients who underwent complete resection; however, with positive resection margins, it was sequenced with chemotherapy [20]. Nonetheless, a low response rate of lung cancer to the standard of care of chemotherapy and radiotherapy was documented [20,24]. A plausible reason was attributed to the heterogeneity of the disease presentation in patients at stage III. These outcomes were further challenged by metastasis resistance to therapies [6,25].

The concept of trying to reduce the tumor burden and opting to scale down its size suggested a pre-surgery therapy to be proposed for patients with lung cancer [18]. This neoadjuvant modality used chemotherapy, which showed a good response in the first years [26]. However, the 4-year survival rate was without statistical significance upon comparison between neoadjuvant chemotherapy and surgery alone administered to patients with lung cancer [26].

The medical community has been investigating molecular and genetic aspects of lung cancer. This line of research lead to the creation of novel targeted therapies and inhibitors to specific mutations [3,27]. This path of personalized medicine, using drugs targeting epidermal growth factor receptor (EGFR) mutations and anaplastic lymphoma kinase (ALK) rearrangements, has been shown to be successful by increasing the survival of patients with lung cancer [3].

Preoperative targeted-therapy induction had promising hopes for better overall survival. The US Food and Drug Administration approved Crizotinib as the first drug to treat NSCLC harboring ALK or ROS-1 fusions [28]. Chen [26] reported that five clinical trials for stage III using targeted therapy agents such as erlotinib and gefitinib managed on one hand to shrink the tumor; nonetheless, on the other hand, the improvement in overall survival relative to neoadjuvant chemotherapy was indefinite [26].

In more recent years, immunotherapy was included as part of neoadjuvant options for non-small cell lung cancer [3]. This breakthrough oncological treatment fights off cancer by targeting tumor cells through enhancing the body’s own defense mechanisms to boost and express a substantial and effective antitumor response [3]. For example, Nivolumab, approved by the FDA in 2015, achieved higher overall survival by administration to advanced non-squamous NSCLC than chemotherapy agent docetaxel [3]. Durvalumab, an anti-PDL1 antibody that serves as an immunocheckpoint inhibitor (ICI) prescribed for non-small cell lung cancer [29], demonstrated longer overall survival. The revolution made by ICI as a treatment modality for NSCLC has supported its incorporation as a first-line approach for advanced-stage disease [29].

In the past few years, there has been a trend toward an increase in patients’ survival probably due to progress in early detection and the introduction of new biological and immunological treatments with better patient-targeted therapy [30]. Other factors that are associated with improved survival are a young age, Caucasian race, female gender, adenocarcinoma subtype, and good performance status [13]. Nevertheless, data of epidemiological and survival aspects of this stage are lacking due to small cohort studies and a short duration of follow-up.

The current study retrospectively evaluated all aspects of Carmel Medical Center experience with this complex group of patients with lung cancer, i.e., stage III NSCLC. The center’s multidisciplinary committee was comprised of specialized medical professionals, e.g., a lung oncologist, pulmonologist, thoracic surgeon, and radiotherapist. The work of this committee and the decision making in real life are explored. In-house cases as well as referred cases were included. The analysis of each case was examined to assess whether the actual treatment protocol aligned with the best recommendations for a diagnostic or follow-up strategy.

The current study focus is on surgical patients who represent a very unique and small subgroup of the heterogenic stage III lung cancer population. The main goal was to revise the path taken along the treatment plan offered to the patients. Another aim was to compare the decisions made during the multidisciplinary committee meetings to guidelines as per the best standard of care at different time points, pre-surgery, surgery, and follow-up.

## 2. Patients and Methods

### 2.1. The Study Design

This study retrospectively evaluated all cases of locally advanced lung cancer that were assessed in the Carmel Medical Center by a multidisciplinary lung cancer committee (comprised of physicians specializing in medical oncology, thoracic surgery, pulmonology, radiotherapy, radiology, pathology, and nuclear medicine) and were referred to surgical intervention after neoadjuvant oncological treatment, from July 2013 to September 2020. The multidisciplinary committee work is by virtue its asset, performed by seeking consensus of the medical team rather than a physician’s preference; the guidelines are rigorously analyzed, examined, and inspected and updates are included for each patient plan [18]. The committee evaluated in-house cases as well as referral cases from other oncological institutions. This study was approved by an Institutional Review Board, study No. 0118-21-CMC, 2021. Consent of patients has been waived due to the retrospective nature of this study.

### 2.2. Clinical Assessment

All patients had undergone Positron Emission Tomography–Computed Tomography (PET-CT) as part of their evaluation and in 21 patients, invasive mediastinal investigation using Endobronchial Ultrasound (EBUS) was performed as part of their clinical staging. All cases were operated on in the Carmel Medical Center cardiothoracic surgical department.

### 2.3. Data Analysis

Descriptive analyses assessing demographic and oncological data as well as treatment patterns were retrieved from medical record files for all patients. The disease diagnosis and clinical staging was reviewed according to the eighth edition of the TNM lung cancer staging system [10]. Neoadjuvant oncological treatment and post-treatment evaluation was analyzed. Detailed surgical data were examined including post-surgical pathological staging. Patients’ follow-up and survival data including overall survival, defined as the duration from surgery to death, were calculated. The Kaplan–Meier method was used to estimate overall survival. The age variable was expressed as the median and mean ± standard deviation. A frequency (*n*) and percentage were employed to describe the patients’ cohorts and clinical pathological staging.

## 3. Results

### 3.1. The Study Population

**Figure 1:**
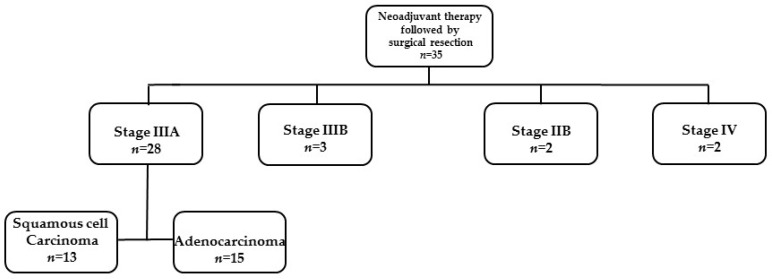
The study population of locally advanced non-small cell lung cancer characterized by clinical disease stage.

From July 2013 to September 2020, thirty-five patients diagnosed with locally advanced non-small cell lung cancer (NSCLC) received neoadjuvant oncological treatment followed by surgical resection in the Carmel Medical Center (Figure 1).

Twenty-eight patients were assessed as clinical stage IIIA. In total, 53.6% of them had adenocarcinoma and thirteen (46.4%) had squamous cell carcinoma. The NCCN guidelines advocate for surgery as part of a multimodality treatment up to stage IIIA; therefore, it was decided to focus on this group of patients.

The other seven patients include three patients assessed as clinical stage IIIB due to a tumor size over 7 cm (T4) and metastatic lymph nodes in one mediastinal station (N2). In these three specific cases, the multidisciplinary committee decided upon surgical intervention due to a good response to neoadjuvant oncological treatment and very limited mediastinal disease. The patients were found to be without evidence of disease at the follow-up from 3.5 to 7 years.

Two patients were evaluated as clinical stage IIB. One patient had a Pancoast tumor with chest wall invasion to the second right rib and the other had limited small cell lung cancer. In those two uncommon cases, the multidisciplinary team supported neoadjuvant oncological treatment prior to surgical intervention. A total of 57 months after the surgical intervention of the patient with a Pancoast tumor, no evidence of disease was detected. The patient with small cell lung cancer had spleen metastasis 6 months after anatomical lung resection (lobectomy) that was treated with radiation. A total of 54 months from the last intervention, the patient was found to be without evidence for lung cancer recurrence.

Two patients were evaluated as clinical stage IV. Those cases were determined as oligometastatic disease with single brain metastasis for each. Overall survival in these cases was 3 months and 36 months after surgery.

The study cohort of patients with clinical stage IIIA lung cancer included twenty-two (78.6%) males and six (21.4%) females with an average age of 64.6 years at surgery (range 47–82 years) (Table 1). Clinical staging was assessed according to the eighth edition of the TNM lung cancer staging system [8].

#### 3.1.1. The Tumor (T) Component

The mean tumor size was 3.9 cm with a median of 3.6 cm (range: 0.8–7.5 cm).

#### 3.1.2. The Node (N) Component

In two patients, no lymph node involvement was found (N0). In four patients, only ipsilateral intra-parenchymal or hilum lymph node involvement was found (N1). In 22 patients, mediastinal lymph node involvement was found (N2). A more detailed analysis of the mediastinal lymph node involvement group showed that 20 patients had only single lymph node station involvement and only 2 patients had multiple-station involvement. N1 + N2 lymph node involvement was observed in 13 patients. In the other 9 patients with N2 disease, no N1 involvement was detected.

### 3.2. Treatment

#### 3.2.1. Neoadjuvant Treatment

The neoadjuvant oncological treatment included chemotherapy with a two-drug combination for all patients. The most common combination was carboplatin and Taxol, which was administered to 20 patients. Radiotherapy was given to 24 patients; 23 patients received a total dose of 60 Gy and 1 patient received 72 Gy. In three patients, preoperative treatment with immunotherapy, either Durvalumab or Pembrolizumab, was added.

All patients, but one, were evaluated at the Nuclear Medicine department, using PET-CT after the completion of neoadjuvant therapy. The only patient that did not go through an imaging PET-CT scan had CT Angiography and an invasive mediastinal investigation using EBUS. In all cases but one, post neoadjuvant disease regression was noted, either in tumor size or in tumor avidity. In one case, the disease was stable without progression.

The median time from neoadjuvant treatment to surgery was 67.5 days (range: 40–230 days).

#### 3.2.2. Surgical Treatment

In terms of surgical approach, 16 patients had video-assisted thoracoscopic surgery (VATS) [3,4] and 10 patients had open thoracotomy. In two cases, the minimally invasive approach had to be converted into an open approach due to massive adhesions in the lung hilum. One conversion was elective and the second was necessary due to bleeding from the pulmonary artery. No other major intraoperative incidents were noted.

Twenty-five lobectomies and three pneumonectomies were performed. In two of the lobectomies, the surgery was extended; in one case, a segmental resection of the lung from the neighboring lobe was added, and a chest wall segment was added in the other case.

Twenty-five patients had an uneventful post-surgical course. Post-surgical complications were noted in three patients and included a septic shock, a broncho-pleural fistula, arterial fibrillation, and lung atelectasis requiring bronchoscopy. The 30-day mortality was nil.

### 3.3. Pathological Staging

The post-surgery pathological staging demonstrated one case of disease progression, eight cases of stable disease, and nineteen cases of disease regression (Table 2). The number of patients enrolled in this study included 28 cases of advanced stage IIIA non-small cell lung cancer, whereas the neoadjuvant oncological treatment followed by surgical resection managed to downstage 67% of them. Importantly, in seven (25%) cases, a pathological complete response was achieved. In these cases, neoadjuvant chemoradiotherapy was administered to six patients and chemoimmunotherapy to one case. In addition, the data show that eight cases were characterized as local disease stage I, and four cases as local disease stage II. Furthermore, out of the three cases initially diagnosed as stage IIIB, none were found to be at this advanced stage upon the completion of this study. Taken together, these results indicate the benefit of neoadjuvant therapy combined with surgical resection as a working protocol reducing NSCLC status to a lower stage.

**Figure 2:**
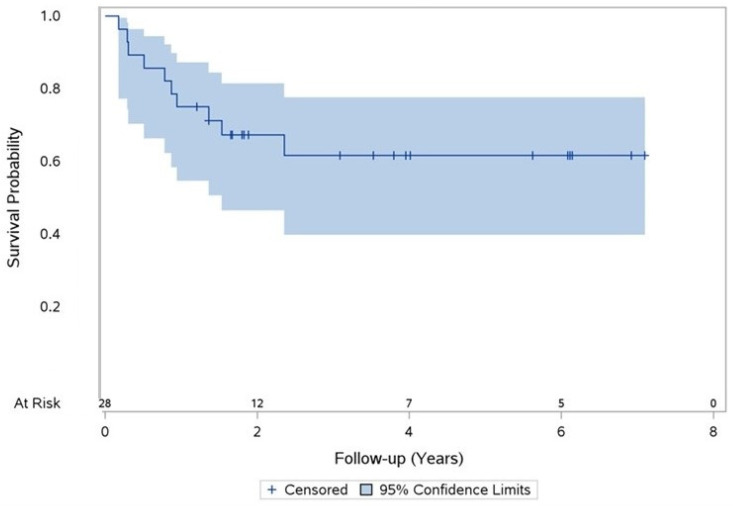
Overall survival statistics for all patients. Product-limit survival estimate with number of subjects at risk.

Ten cases of mortality from any cause during the study follow-up were documented. The 2-year overall survival rate was 67% and the 5-year overall survival rate was 62% (Figure 2).

Disease recurrence was documented in ten (36%) patients out of which seven patients had distant disease recurrence (e.g., liver, brain, adrenal gland, bones, and lymph nodes) and three patients had a combination of loco-regional and distant recurrence (e.g., contralateral lung and pleura).

## 4. Discussion

The aim of this study was to evaluate the real-life results of a multimodality treatment approach to patients with locally advanced lung cancer. During the time frame of seven years, of which retrospective medical records’ data were collected, the lung cancer fast-developing research has been adjusted and updated and probably affected the administered therapies. It was evident by the fact that most patients in the cohort had a neoadjuvant chemoradiation combination, where a couple different agents were employed, though a handful received immunotherapeutic drugs. Along the studied period, the institute’s multidisciplinary tumor board team in the Carmel Medical Center assisted in offering appropriate treatment decisions according to current available diagnostics for this heterogamous population. Each individual case was fit with an optimal treatment plan to be delivered. This shows the variety and non-uniformity that exists in the field, where appropriate considerations take place. An advantage of this multidisciplinary committee approach is recognized through its numerous members’ expertise in each field [31]. In addition, the medical professional specialists have experience and knowledge of the patient condition along the duration of the protocols’ steps and follow-up, which contributes to the best standard of care [31,32]. One of the roles of the tumor board multidisciplinary committee is to consider the possible risks related to the diverse therapies such as toxicity adverse events of chemotherapy, radiotherapy, and immunotherapy [32]. The added value of the committee meetings is the sharing of knowledge for the sake of the patients’ benefit.

Although the combination of neoadjuvant therapy followed by surgery for stage IIIA is recommended in the NCCN guidelines for suitable patients [11] by reason of a documented increase in survival [16], in real-life experience, it is not the routine strategy for the majority of patients with stage III lung cancer. Literature reviews indicate that most patients in this stage (IIIA) will be referred to oncological treatment only and surgery will be abandoned [15,33,34].

Clinical studies that evaluate multimodality treatment for locally advanced lung cancer are relatively few. The small number of patients with stage IIIA treated with multimodality therapy including surgery is probably low due to several reasons. Some could be patient-related reasons such as low performance status, and inability to endure surgery due to a medical or mental condition. Other reasons could reflect the patient’s physician preference of oncological treatment. The necessity for a cooperative multidisciplinary team that evaluates the patient status before and during all stages of treatment is a demanding requirement, which is needed for this kind of patient-oriented treatment in today’s medicine. The role of the multidisciplinary committee is especially important in light of the rapid changes and developments taking place in lung cancer treatment these days.

The endpoint result of the present study demonstrated a relatively high overall survival in two and five years after the combined oncological–surgical treatment concluded. These results reflect a suitable selection of patients for this line of multimodality treatment. An early indication for this good outcome was seen in the pathological complete response rate (25%) after surgery. The pathological complete response is a factor that is growingly used today as a surrogate endpoint for survival. It is as high as seen in recently published studies of modern chemo-immunological combination systemic therapies for patients with operable lung cancer [35].

The surgical results can attest to the feasibility of resection surgery after chemoradiation treatment as reported in other studies [36,37]. The ability to perform surgery in most cases with a minimally invasive approach and low conversion rate, combined with R0 pathological resection in all cases and a low complication rate, represents the important value of a dedicated oncological thoracic surgical team. Although the present study cohort includes a small number of pneumonectomies, complications were not encountered in this subgroup [38].

The local disease control as represented in the described disease recurrence patterns (seven cases of distant recurrence versus three cases of loco-regional and distant recurrence) is an indication for the overall good local control achieved in this multimodality treatment approach, with a crucial part attributable to the resection procedure.

West et al. [19] have reported that 70% of patients bearing operable NSCLC tumors experience recurrence following surgery. Advanced loco-regional cancer spread in the form of metastasis was detected both at loco-regional and distant sites [19]. This called for a broader approach to complement the surgery step, by adding post-surgery adjuvant therapy.

The low response rate attributed to lung cancer therapies arises from the fact that metastatic tumors may bear resistance [39] to chemotherapy and radiation. This may be due to the altered genetic material of cancer cells, e.g., mutated oncogenes [39].

Moreover, passing the blood–brain barrier could be achieved only by a few recently developed drugs/agents such as entrectinib [40] that are able to reach metastatic CNS lesions of an NSCLC origin.

Wang et al. [27] have reviewed novel immuno- and targeted therapies developed to aim the resistance posed by advanced NSCLC to previous decades’ drugs. Current perspectives of patients with advanced NSCLC of neoadjuvant targeted therapies available as the standard of care for resectable stage III tumors were reviewed by Lee [41]. The main issue is posed by metastatic recurrence, which could allude to a poor survival rate. Therefore, the adjuvant therapy importance is also emphasized, as part of comprehensive perioperative strategies.

Forde et al. [21] reported that a complete response was achieved in 24% of patients with NSCLC who had been treated with a neoadjuvant protocol composed of Nivolumab as an immunotherapy agent in addition to chemotherapy. This protocol showed an advantage over chemotherapy alone in a median event-free survival of 31 months compared to 20.8 months. Nivolumab is an example of an approved PD-1 inhibitor involved in neoadjuvant administration with chemotherapy for patients bearing stage III NSCLC [42]. Of note, similarly, Zappa [3] reviewed the management of lung cancer, indicating the advantage of personalized, custom-tailored medicine through targeted therapies in addition to novel immunotherapy in extending the patients’ survival. Nonetheless, even potent targeted therapies are vulnerable to drug-tolerant tumor cells; therefore, ongoing research continues to investigate and study ways to overcome and clinically treat NSCLC [25].

Daly et al. [18] reviewed multiple options for managing NSCLC heterogeneous disease. The suggested guidelines list criteria aiming to match treatment according to each individual condition. The use of an expert panel hosting medical oncologists, thoracic surgeons, radiation oncologists, and pulmonary oncologists is advised while analyzing each case in detail to enable planning of perioperative protocol recommendations; hence, these include neoadjuvant pre-surgery treatment, surgery resection, and post-surgery, such as concurrent adjuvant chemo- and radiotherapies.

Perioperative treatment is the most novel approach presently applied for patients with lung cancer. The medical community has acknowledged the need to advocate for the patients’ best care along the different clinical steps. This is conducted first through a complete initial diagnosis followed by the suggestion of neoadjuvant therapy, which could benefit the patient intended for undergoing surgery and later the postoperative adjuvant therapy [43].

Christopoulos [44] summarized the updated paradigm of perioperative treatment in managing resectable non-small cell lung cancer. The findings of that publication demonstrated the benefit of a perioperative strategy. The combination of chemoimmunotherapy administered for three cycles as the neoadjuvant step, surgical resection, and adjuvant programmed cell death ligand 1 inhibition for a duration of one year significantly augmented the event-free survival. The efficacy of perioperative treatment as a whole was shown to improve overall survival compared with either pure adjuvant or pure neoadjuvant approaches. The medical objective to achieve a pathologic complete response is enabled by the perioperative strategy that allows postoperative adjustment of an intensified therapy [44].

The current study has an inherent limitation due to its selection bias of patients, small sample size, and inconsistent treatment regimens, both oncological neoadjuvant and surgical, which are the result of a retrospective data collection of a relatively highly selective group of patients. Yet, it is important to show the good results of this combined treatment approach for suitable patients with NSCLC.

## 5. Conclusions

It can be concluded that in this real-life small-number highly selective patient study, there is a benefit for the combination of oncological neoadjuvant chemoradiation followed by surgery treatment for patients with resectable locally advanced lung cancer. Future studies should adhere to updated developments to provide complete perioperative therapy aiming to maximize antitumor strategies, while minimizing adverse events. Close monitoring is advised to detect early post-surgery changes that would indicate additional adjuvant therapy. Encompassing all possible modalities to extend patients’ survival and quality of life should be the beacon leading the way both for the ongoing research to overcome tumor resistance as well as the multidisciplinary professionals’ committee role in advising therapy approaches.

## Figures and Tables

**Table 1:** Demographic and clinical characteristics of the study participants (*n* = 28).

Characteristic	Male	Female	Cohort
Number of patients, *n* (%)	22 (78.6)	6 (21.4)	28 (100)
Age (years),			
Median	63.5	67.5	64.6
(Mean ± SD ^1^)	(64.5 ± 17.5)	(59 ± 10.5)	(64.5 ± 17.5)
Adenocarcinoma, *n*	11	4	15
Squamous cell carcinoma, *n*	11	2	13
Tumor size, cm ^1^ (mean)	4.4	2.5	3.9
Lymph node involvement			
N0	2	0	2
N1	2	2	4
N2	18	4	22

^1^ Abbreviations: SD, standard deviation; cm, centimeters.

**Table 2:** Post-surgery pathological staging of the study participants (*n* = 28).

Pathological Staging	Total
Pathological complete response, *n* ^1^ (%)	7 (25%)
Local disease stage I, *n* (%)	8 (29%)
Stage IA1, *n*	2
Stage IA2, *n*	4
Stage IA3, *n*	1
Stage IB, *n*	1
Local disease stage II, *n* (%)	4 (14%)
Stage IIA, *n*	2
Stage IIB, *n*	2
Locally advanced disease stage III, *n* (%)	8 (29%)
Stage IIA, *n*	8
Stage IIB, *n*	0
Metastatic disease stage IV, *n* (%)	1 (3%)

^1^ *n* denotes frequency, % represents percent of total.

## Data Availability

The data presented in this study are available upon request from the corresponding authors.
